# The emerging role of molecular pathology in directing the systemic treatment of endometrial cancer

**DOI:** 10.1177/17588359211035959

**Published:** 2021-08-14

**Authors:** Amy Jamieson, Tjalling Bosse, Jessica N. McAlpine

**Affiliations:** Department of Gynaecology and Obstetrics, Division of Gynaecologic Oncology, University of British Columbia, Vancouver, BC, Canada; Department of Pathology, Leiden University, Leiden, The Netherlands; Department of Gynaecology and Obstetrics, Division of Gynaecologic Oncology, University of British Columbia, 2775 Laurel St, Vancouver, BC V6L-1Z5, Canada

**Keywords:** chemotherapy, endometrial cancer, molecular oncology, molecular testing, targeted therapy

## Abstract

Following the discovery of the four molecular subtypes of endometrial cancer (EC) by The Cancer Genome Atlas (TCGA) in 2013, subsequent studies used surrogate markers to develop and validate a clinically relevant EC classification tool to recapitulate TCGA subtypes. Molecular classification combines focused sequencing (*POLE*) and immunohistochemistry (mismatch repair and p53 proteins) to assign patients with EC to one of four molecular subtypes: *POLE*mut, MMRd, p53abn and NSMP (no specific molecular profile). Unlike histopathological evaluation, the molecular subtyping of EC offers an objective and reproducible classification system that has been shown to have prognostic value and therapeutic implications. It is an exciting time in EC care where we have moved beyond treatment based on histomorphology alone, and molecular classification will now finally allow assessment of treatment efficacy within biologically similar tumours. It is now recommended that molecular classification should be considered for all ECs, and should be performed routinely in all high grade tumours. It is also recommended to incorporate molecular classification into standard pathology reporting and treatment decision-making algorithms. In this review, we will discuss how the molecular classification of EC can be used to guide both conventional and targeted therapy in this new molecular era.

## Introduction

Globally, the incidence of endometrial cancer (EC) has increased significantly by 0.69% per year between 1990 and 2019,^1^ and mortality continues to rise. Patients with advanced and recurrent EC represent a major therapeutic challenge, with 5-year overall survival rates of only 17% in patients with distant disease.^2^ Approximately 80% of women with early-stage EC have a favourable prognosis, with 5-year overall survival rates of 95%.^3^ The remaining 20% of women with early-stage disease have one or more high risk features associated with an increased risk of cancer-related death. A major diagnostic challenge is identifying which patients with early-stage EC have low-risk disease with a risk of recurrence <5%, and thus can be managed by surgery alone, in contrast to patients with high-risk disease who need adjuvant therapy.

For decades, the EC risk stratification used to guide treatment has been based largely on histological type, grade and stage. Both histotype and grade assignment have been shown to be poorly reproducible even amongst expert pathologists, with no agreement in histotype diagnosis in one-third of cases.^4,5^ Stage assignment is generally considered more objective and consistent; however, pathology review of all patients eligible for the PORTEC-3 trial for high risk EC found not only significant disagreement in grade and histotype, but also in stage- and risk-group-defining criteria such as cervical stromal invasion, myometrial invasion and lymphovascular invasion (LVI).^6^ As such, a risk stratification where all three of the critical parameters are inconsistent leads to an imprecise estimation of the risk of recurrence and death, which subsequently results in both the over- and under-treatment of many women.

What is also concerning is the significant variation in clinical practice that is currently observed in administering adjuvant therapy in EC.^7^ A recent National Cancer Database review showed out of 19,594 EC patients who met the national guidelines criteria for adjuvant radiation, 47% did not receive radiation, which was associated with worse overall survival. Omission of adjuvant radiation was more common among African-American, Hispanic and Asian patients, and patients with lower household income, lack of health insurance and treatment at non-academic hospitals.^8^ This highlights the need for an objective EC classification system with fair and consistent delivery of care.

We are now in an exciting era in EC research and clinical care, moving to a subtype specific approach proven successful in other disease sites. In 2013, The Cancer Genome Atlas (TCGA) discovered four molecular subtypes of EC-based genomic architecture, with each group having distinct clinical outcomes; *POLE* ultra-mutated, microsatellite instability hypermutated, copy-number low and copy-number high.^9^ Following this discovery, two groups developed and validated a molecular classification tool using surrogate markers to recapitulate TCGA subtypes using more pragmatic, cost-effective and clinically applicable methods.^10–14^ This classifier uses a combination of focused sequencing to identify mutations in DNA polymerase epsilon (*POLE*) and immunohistochemistry (IHC) for mismatch repair proteins and p53, yielding four molecular subtypes: *POLE*mut, mismatch repair deficient (MMRd), p53abn and NSMP (no specific molecular profile, with normal p53 expression/p53wt). The molecular classification of EC has been shown to be highly reproducible among pathologists, with high concordance between the preoperative biopsy and final hysterectomy specimens, as well as high interlaboratory concordance.^15–17^ The prognostic value of molecular classification has been demonstrated repeatedly, across unselected population-based series, clinical trials, and even narrowly defined age- or histotype-stratified subsets.^18–21^ What has been most promising, however, is the recent data supporting the predictive implications of molecular subtype assignment. This new reproducible and objective classification system creates a much needed framework to approach treatment decisions in both conventional and targeted therapy. Given the new recommendations for integration of molecular parameters in to standard pathology reporting in the 2020 5th edition of the WHO Female Genital Tumours, and the integration of molecular classification into the 2020 ESGO/ESTRO/ESP risk stratification and treatment algorithms, it is imperative that clinicians are aware of the rationale for molecular subtype-specific care.^22^

In this review, we will discuss how the molecular classification of EC can be used to help guide systemic treatment in this new molecular era.

## MMRd endometrial cancer

The MMRd/microsatellite unstable molecular subtype accounts for 25–30% of all ECs and they have an intermediate prognosis.^9–14^ These tumours have loss of DNA mismatch repair, which results in a high mutational burden (‘hypermutated’), exceeding >10 mutations per megabase.^9^ Mismatch repair deficiency can be identified through microsatellite instability testing, or testing for the loss of expression of one or more mismatch repair proteins (MLH1, PMS2, MSH6, PMS2). Approximately 90% of MLH1/PMS2 loss is due to somatic *MLH1* promotor hypermethylation, and germline mutations in one of the mismatch repair genes, termed Lynch syndrome, accounts for approximately 10% of MMRd ECs and 3% of all ECs.^23,24^ Post *et al*.^24^ also recently assessed the prognostic value of Lynch Syndrome within MMRd EC and found a trend towards improved recurrence free survival in patients with Lynch Syndrome compared with *MLH1* hypermethylation.

The ‘hypermutated’ MMRd and ‘ultramutated’ *POLE*mut ECs, are known to be highly immunogenic tumours. Previous work has demonstrated cancers that have a high mutational burden have a substantially increased production of tumour mutated antigens (neoantigens), which correlates significantly with improved patient survival.^25^ The increased neoantigens results in a high abundance of tumour-infiltrating lymphocytes (TIL), in particular CD8+ cytotoxic T cells, with an upregulated T-cell mediated antitumour response.^25–27^ Cancers can develop mechanisms of immune escape, largely through upregulation of inhibitory immune checkpoint receptors, such as programmed death-ligand 1 (PD-L1), which subsequently block activated T-cell-mediated tumour cell death.^27^ These immune checkpoint interactions between tumour cell PD-L1 receptors and programmed death protein 1 (PD-1) on T-cells can be blocked by the use of antibodies, thus making these tumours susceptible to reactivation of the immune response when treated with immune checkpoint blockade therapy.

### Conventional adjuvant therapy

The role of adjuvant chemotherapy in MMRd EC has been questioned recently by the molecular analysis of PORTEC-3. This trial assessed chemotherapy used in addition to adjuvant radiation in high-risk EC. The molecular analysis found no benefit with the addition of chemotherapy in the MMRd group, with the 5-year overall survival 84% in the radiation only group *versus* 79% in the chemoradiation group (p = 0.445).^28^ Adjuvant radiation on the other hand, may play a more important role in MMRd EC, compared with other EC molecular subtypes. Pre-clinical work has shown increased sensitivity to radiation in MSH2 deficient cell lines.^29^ In a review of 128 patients with stage Ib/II grade 3 endometrioid endometrial cancer, Reijnen *et al.* showed that adjuvant radiation was associated with improved disease specific survival in the MMRd group, but not in MMR-proficient cases.^30^ A more recent study compared adjuvant chemotherapy and radiation with chemotherapy alone in advanced MSI-high EC, and found an improved progression-free survival with the addition of radiation.^31^ This evidence suggesting MMRd EC may have an increased sensitivity to radiation needs to be validated in prospective studies.

### Immune checkpoint blockade therapy

Pembrolizumab (anti-PD-1) was the first immune checkpoint inhibitor shown to have favourable objective response rates (ORR) in metastatic or recurrent MMRd colorectal and non-colorectal cancers.^32,33^ This subsequently led to the United States (US) Food and Drug Administration (FDA) approval in 2017 of pembrolizumab in unresectable or metastatic MSI-H/MMRd solid tumours. The ORR of pembrolizumab in the MMRd EC cohorts in these studies were a striking 53–57%. Several other single agent immune checkpoint inhibitors have since been studied in advanced or recurrent EC, including nivolumab (anti-PD-1), avelumab (anti-PD-L1), durvalumab (anti-PD-L1) and dostarlimab (anti-PD-1), with the ORR in MMRd cohorts being 25%, 27%, 43% and 42%, respectively.^34–37^

**Figure 1.**
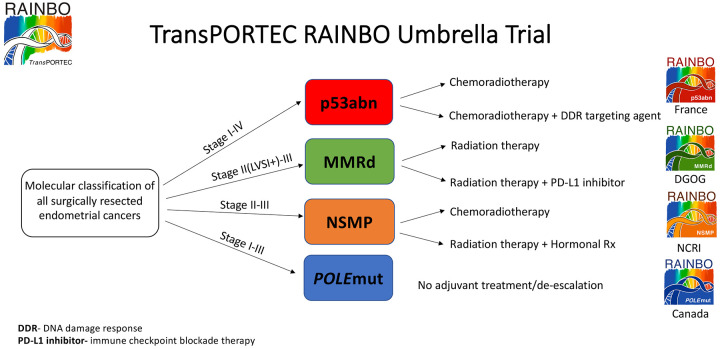
The planned treatment arms for the TransPORTEC RAINBO program of clinical trials. DDR, DNA damage response; PD-L1, programmed death-ligand 1; POLE, polymerase epsilon; MMRd, mismatch repair deficient; NSMP, no specific molecular profile; RAINBO, refining adjuvant treatment in endometrial cancer based on molecular profile.

To date, the efficacy of immune checkpoint inhibitors has been assessed in EC only in the advanced or recurrent setting, with at least one prior line of platinum-based chemotherapy. Whether patients with MMRd EC would benefit from the addition of immunotherapy to conventional adjuvant therapy regimes remains unknown. The TransPORTEC refining adjuvant treatment in endometrial cancer based on molecular profile (RAINBO) program of clinical trials plans to randomise patients with stage II/III MMRd EC to radiation *versus* radiation plus an immune checkpoint inhibitor (Green-MMRd trial; Figure 1). Similarly, the ADELE trial (adjuvant Tislelizumab plus chemotherapy after chemoradiation in high risk endometrial cancer: the ADELE study) plans to assess the efficacy of an immune checkpoint inhibitor used in addition to standard chemoradiation and chemotherapy in patients with high-risk MMRd EC.

### Immune checkpoint blockade combinations

KEYNOTE-146 is a phase IB/II study of pembrolizumab in combination with lenvatinib – a multikinase inhibitor that targets vascular endothelial growth factor receptors 1–3 in selected advanced solid tumours. The EC cohort (*n* = 108) showed an ORR of 64% in the MMRd group and 36% in the MMR-proficient group.^38^ This led to the FDA approval of pembrolizumab plus lenvatinib in MMR-proficient EC patients who have disease progression following prior systemic therapy in 2019. KEYNOTE-775 is a phase III trial of pembrolizumab plus lenvatinib compared with physician’s choice chemotherapy (doxorubicin or paclitaxel) in advanced or recurrent EC in patients with at least one prior line of platinum-based chemotherapy. The results were presented at the recent Society of Gynecologic Oncology (SGO) annual meeting 2021 with significant improvement in both progression-free survival (7.2 months *versus* 3.8 months) and overall survival (18.3 months *versus* 11.4 months) in the pembrolizumab plus lenvatinib arm compared with chemotherapy in all comers (both MMR-proficient and MMRd EC).^39^ What remains to be determined is how much benefit is gained by the addition of lenvatinib to pembrolizumab within MMRd tumours and whether the additional toxicity is warranted in this group. There is another recently completed phase III study assessing this pembrolizumab plus lenvatinib combination but in first-line treatment compared with first-line carboplatin and paclitaxel in patients with advanced or recurrent EC (LEAP-001/ENGOT-en9).

The combination of nivolumab and cabozantinib (multiple tyrosine kinase inhibitor) in recurrent EC with prior systemic therapy was presented at ASCO 2020. This combination resulted in an ORR of 25% and a clinic benefit of 69% in this heavily pre-treated group.^40^ A subgroup analysis included 21 patients post progression on immune checkpoint blockade therapy alone, and the nivolumab and cabozantinib combination resulted in an ORR in 5/21 and stable disease in 12/21, suggesting immune blockade re-challenge combined with anti-angiogenics may be a way to overcome resistance. There was also signal of activity with combination therapy in recurrent carcinosarcoma, with ORR in 1/9 and stable disease in 4/9 patients.

There are also currently three clinical trials ongoing assessing the efficacy of immune checkpoint inhibition combined with first-line carboplatin and paclitaxel chemotherapy in advanced or recurrent EC. This includes the AtTEnd trial (AtTEnd/ENGOT-en7), which is using atezolizumab, RUBY (ENGOT-EN6/NSGO-RUBY), which is using dostarlimab and NRG-GY018 using pembrolizumab. The results of all of these ongoing and upcoming trials will help clarify the optimal timing of administration of immunotherapy in MMRd EC that is, whether it be adjuvant use in high-risk patients, combined use with first line platinum-based chemotherapy, combination use with targeted therapy or reserved for patients who have failed prior systemic therapy. Another important question is which patients with other EC molecular subtypes in addition to MMRd may benefit from immunotherapy. Two studies have assessed the immune response in large cohorts of molecularly classified ECs, and both groups found heterogeneity of TIL across EC subtypes with a proportion of both p53abn and NSMP subtypes surprisingly immune-rich.^41,42^ This suggests molecular classification used alone to direct use of immune checkpoint blockade therapy may miss a significant number of EC patients who may benefit, and further research to identify biomarkers that can accurately predict response to immunotherapy is required.

## p53abn endometrial cancer

p53abn EC represents the most aggressive and lethal molecular subtype, and although it accounts for only approximately 15% of all EC cases, it is responsible for 50–70% of EC mortality.^9–14,18–21^ The ECs in this molecular subtype were characterised by TCGA as having a very high number of somatic copy number alterations, low mutation rate and ubiquitous *TP53* mutations.^9^ These tumours are now identified by a more pragmatic method of mutant-pattern p53 IHC staining, which has been shown to be an excellent surrogate maker for *TP53* mutational status as determined by sequencing in EC biopsies.^43^ A large majority of serous carcinomas are p53abn; however, p53abn EC is seen across all EC histological types. The proportion of p53abn tumours for each histotype was assessed recently using pooled data from several EC cohorts that have been classified molecularly as follows; serous carcinoma 93%, carcinosarcoma 85%, clear cell carcinoma 38%, grade 3 endometrioid EC 22% and grade 1–2 endometrioid EC 5%.^7,9–14,18–21,44^

### Conventional adjuvant therapy

The importance of conventional platinum-based chemotherapy in p53abn EC was demonstrated with the retrospective molecular analysis of PORTEC-3 trial for high-risk EC. This showed that patients with p53abn EC had significantly improved outcomes when chemotherapy was used in addition to pelvic radiation, with 5-year recurrence-free survival of 59% with chemotherapy and radiation *versus* 36% with radiation alone (*p* = 0.019).^28^ This significant benefit from the addition of chemotherapy in the p53abn group was not observed in the other molecular subtypes. The overall test for interaction between molecular subgroups and treatment arm did not reach significance; however, the PORTEC-3 clinical trial was not originally powered for analysis by molecular subgroup. This signal of benefit with conventional chemotherapy observed within p53abn EC is promising. Although historical EC studies have shown very mixed results in terms of adjuvant platinum-based chemotherapy response, the ability to easily stratify (through p53 IHC) and identify a subset of women who appear to benefit will enable improved design of future trials. Further, this is a subset of women in whom better treatment options and opportunities to improve survival statistics are desperately needed. The evidence that p53abn molecular subtype is found in up to 5% of grade 1 and 2 endometrioid endometrial carcinomas^7,45^ highlights why it is important that molecular features are integrated into pathology classification and stratification of care. Patients with stage I and grade 1–2 endometrioid EC without LVI would otherwise be considered low risk by historical EC risk stratification systems, and might be treated by surgery alone. Although further work is needed to better understand this subgroup of cancers, the concern is without p53 stratification they may be undertreated, representing a missed opportunity for curative adjuvant therapy.

### Homologous recombination deficiency

Despite this benefit from conventional chemotherapy, we know the survival outcomes for patients with p53abn EC are poor and we urgently need therapeutic advances to improve outcomes for these patients. TCGA demonstrated several molecular similarities between *TP53* mutated EC and both high-grade serous tubo-ovarian cancer (HGSOC) and basal-like breast cancer.^9^ Defects in the homologous recombination repair pathway are common in HGSOC and basal-like breast cancers, found in 55% and 46% respectively.^46,47^ Homologous recombination is essential for the repair of double-stranded DNA breaks, and tumours with homologous recombination deficiency (HRD) are known to be more responsive to both platinum-based chemotherapy and poly (ADP-ribose) polymerase (PARP) inhibitors. The prevalence of HRD in p53abn EC is currently unknown. De Jonge *et al.* assessed HRD in a small group of ECs using functional RAD51assessment and found HRD in 46% of p53abn EC,^48^ although this may be an over representation in this highly selected group. Another study assessed HRD in 19 uterine serous carcinomas using OncoScan SNP arrays to calculate HRD scores and found 53% had an HRD phenotype.^49^ However, another group found only 15% of copy-number high EC from TCGA dataset had mutational signatures that were HRD related.^50^ Siedel *et al.* used the Myriad HRD assay on two EC cohorts and found a cut-off HRD score ⩾4 was associated with worse survival, and the median HRD scores for endometrioid EC, mixed serous and endometrioid EC, and serous EC were 3, 15.5 and 28.5 respectively.^51^ The worse survival outcomes for high HRD score EC differs from HGSOC and basal-like breast cancer, where HRD high tumours show improved outcomes in both tumour types. This same group also determined the HRD scores of 12 EC cell lines. The three cell lines with highest HRD score were all *TP53* mutated and all showed statistically significant increased sensitivity to cisplatin, paclitaxel and olaparib, compared with the low HRD score cell lines.

NRG-GY012 is a phase II, three-arm study in recurrent and metastatic EC assessing the PARP inhibitor olaparib in combination with the anti-angiogenic cediranib, compared with both agents used alone in recurrent, refractory or metastatic EC. The results, presented at the SGO annual meeting 2021, showed the combination of olaparib and cediranib demonstrated modest efficacy, but was not significantly different compared with cediranib alone. Single agent olaparib was not effective in this patient population and the molecular analysis including subtype distribution and HRD scores from this trial cohort are still pending.^52^

DOMEC (durvalumab and olaparib in metastatic or recurrent endometrial cancer) is another ongoing phase II study looking at olaparib combined with the immune checkpoint inhibitor durvalumab in recurrent or metastatic EC. The efficacy of PARP inhibitors used in combination with adjuvant first line chemotherapy in p53abn EC will also be assessed in CAN-STAMP (assessing front line chemotherapy +/− targeted therapy *versus* conventional chemoradiation in early and late stage serous or p53abn endometrial cancer). The TransPORTEC RAINBO program of clinical trials plans to assess adjuvant chemoradiation with or without a DNA damage response (DDR) targeting agent in p53abn EC (Red-p53abn trial, Figure 1).

### Human epidermal growth factor 2

Another systemic treatment opportunity for p53abn EC is anti-human epidermal growth factor 2 (HER2) therapy. HER2 overexpression and/or amplification has important prognostic and therapeutic implications in breast and gastric cancer. HER2 overexpression and/or amplification is seen in EC, with variable reported frequencies. Unlike breast and gastric cancer, there is no standardised scoring system of HER2 in EC and, consequently, what is reported as ‘HER2 positive EC’ varies considerably between studies and makes it difficult to interpret the literature. Buza *et al.*, however, recently reported on a proposed serous EC specific HER2 scoring system with moderate to substantial interobserver agreement among gynaecologic pathologists.^53^ Vermij *et al.* also recently evaluated a HER2 IHC scoring system on 78 p53abn ECs of all histotypes, in order to establish an optimal diagnostic HER2 testing algorithm for p53abn EC.^54^ They also found substantial interobserver agreement with this molecular subtype directed HER2 scoring. This work provides an important step towards refining inclusion criteria for anti-HER2 therapy EC clinical trials.

Despite disappointing results in a previous clinical trial assessing single agent trastuzumab in previously treated, non-stratified ECs,^55^ there is now data demonstrating improved outcomes within serous ECs. The phase II trial from Fader *et al*.^56,57^ showed the addition of trastuzumab to carboplatin and paclitaxel chemotherapy for women with advanced or recurrent uterine serous carcinoma, that were HER2 positive (3+ IHC or 2+IHC with amplification confirmed on fluorescence *in situ* hybridisation) significantly improved both progression free survival (12.6 months *versus* 8.0 months) and overall survival (29.6 months *versus* 24.4 months) compared with chemotherapy alone. The largest benefit was seen in patients with stage III/IV disease, with a progression free survival of 17.9 months with chemotherapy plus trastuzumab *versus* 9.3 months with chemotherapy alone. Molecular stratification has not been performed on this series.

HER2 status was recently assessed retrospectively in PORTEC-3 cases that had undergone molecular classification. They found 25% of the p53abn molecular subtype were HER2 positive (moderate or strong HER2 IHC staining with confirmation of amplification on dual *in situ* hybridisation). The correlation between p53abn and HER2 status was significantly stronger than between serous histology and HER2 status.^58^ The HER2 positive cases were seen across all p53abn histotypes; including serous, endometrioid, and clear cell carcinomas. Similar findings were also recently reported in a cohort of 238 p53abn ECs which underwent sequencing of >400 cancer related genes. *ERBB2* alterations were found in 21% of cases, and the frequency of *ERBB2* amplification did not differ between histological types.^59^ Both of these studies highlight the importance of molecular subtype directed clinical trials rather than histotype directed, to maximise the number of patients who may derive clinical benefit from anti-HER2 therapy.

### Anti-angiogenic agents

Anti-angiogenic agents have been successful in many cancer types but have generally shown disappointing results in EC to date.^60^ Single agent bevacizumab (a monoclonal antibody against vascular endothelial growth factor-A) was assessed in recurrent or persistent EC after at least one prior line of chemotherapy, with 13.5% of patients demonstrating a clinical response (one complete response and six partial).^61^ The MITO END-2 trial compared carboplatin, paclitaxel and bevacizumab in 108 patients with advanced or recurrent EC, with chemotherapy alone. Bevacizumab combined with chemotherapy failed to demonstrate a significant improvement in progression-free survival; however, it did show a significant increase in 6-month disease control rate (70.4% *versus* 90.7%).^62^ GOG-86P was a recent randomised three-arm trial assessing the addition of either bevacizumab, temsirolimus or ixabepilone, with standard paclitaxel and carboplatin in advanced or recurrent EC. Progression free survival was not increased in any of the three experimental arms compared with historical controls. In a *post hoc* analysis, assessment of *TP53* mutation status showed that women with *TP53* mutant EC had both improved progression free- and overall survival when treated with bevacizumab and chemotherapy, whereas women with *TP53* wild-type tumours showed no difference in outcomes.^63^ The authors concluded p53/*TP53* could be used as a biomarker to help predict patients with EC more likely to respond to bevacizumab treatment.

### Other therapeutic opportunities for p53abn endometrial cancer

Wee1 is a protein kinase that regulates the G2 checkpoint and prevents entry into mitosis in response to DNA damage. This G2 checkpoint is important for *TP53* mutant cells that have impaired G1 checkpoint repair and therefore rely on the G2 pathway for DNA repair.^64^ A phase II study looking at the use of single agent Wee1 inhibitor adavosertib in patients with recurrent uterine serous carcinoma and at least one prior line of platinum-based chemotherapy was presented at the ASCO 2020 meeting. They found an objective response rate of 29.4% and a clinical benefit rate of 50%, which is higher than observed with monotherapy in other disease sites.^65^ ADAGIO (a study of adavosertib as treatment of uterine serous carcinoma) is a larger phase II study looking at single agent adavosertib in the same patient population.

## NSMP endometrial cancer

Deriving from ‘copy number low’ TCGA subtype, NSMP ECs are characterised by a low number of somatic copy number alterations, low mutational burden and high levels of oestrogen and progesterone receptor (ER/PR) expression. As implied by their name ‘no specific molecular profile’ they are defined by their lack of pathogenic *POLE* mutations, mismatch repair or p53 abnormalities. They are the most common molecular subtype, accounting for approximately half of all ECs, with an intermediate prognosis. They are arguably the most challenging molecular subtype, given the lack of predictive biomarkers that can identify patients within this large heterogeneous group that have a higher risk of disease recurrence, and may benefit from more aggressive therapy. One potential stratification biomarker is β-catenin (*CTNNB1*) mutation status. Mutations in exon 3 of *CTNNB1* are present in 52% of NSMP ECs and studies have shown low-grade endometrioid ECs that have a *CTNNB1* mutation are a more aggressive subset with a higher risk of disease recurrence.^9,14,66^ L1-cell adhesion molecule (L1CAM) overexpression has also been shown to be an independent prognostic marker for distant recurrence and overall survival within NSMP ECs.^14,67^ Immune profiling of 695 ECs that had been molecularly classified demonstrated 36% of NSMP tumours had high immune cell infiltrate, and intraepithelial CD8+ cell density was a significant predictor of recurrence, and thus can be used to refine prognostication in this molecular subtype. Whilst we await further molecular refinement within NSMP EC, grade, stage, LVI and histotype will continue to play a role in determining adjuvant therapy for this subtype.

### Hormonal therapy

Despite being used for several decades in ER-/PR-positive EC, mostly in the recurrent/metastatic setting, the role of hormonal therapy in EC remains poorly defined. In contrast to breast cancer, hormonal therapy in ER/PR positive EC is effective only in a minority of women, with response rates of approximately 22% to first-line hormonal therapy in advanced disease.^68^ Interpreting the current hormonal therapy literature in EC is challenging due to the unselected EC cohorts included in historic trials, different response criteria used in different studies, different hormonal treatment regimes and different cut offs used to define ER/ PR positive EC.

Another priority is determining which ECs will respond to hormonal therapy, beyond IHC analysis for ER and/or PR expression. In breast cancer, hormonal therapy has a very important role for patients with ER-positive disease, but not all patients who have positive ER expression on IHC will respond to therapy. A functional ER score based on activity from mRNA levels of ER pathway specific target genes was proposed as a more reliable method of predicting response to hormonal treatment in breast cancer compared with standard ER IHC staining. They found one third of a cohort of ER positive breast cancers had a functionally inactive ER pathway score which was associated significantly with non-responding hormone status.^69^ Studies such as this would be extremely valuable in EC and may explain why response rates to hormonal agents in ER/PR positive EC have been disappointingly low. Given the high levels of ER and/or PR expression seen in NSMP EC, the TransPORTEC RAINBO program of clinical trials plans to randomise NSMP EC to adjuvant chemoradiation *versus* radiation plus hormonal therapy (Orange-NSMP trial, Figure 1).

### Hormonal therapy plus targeted agents

Hormonal therapy combined with targeted therapeutic agents known to disrupt ER pathways appears to be an effective mechanism of increasing response rates. One example is the use of mTOR inhibitors. Mutations in the PI3K/Akt/mTOR pathway are frequently seen in EC, and specific to NSMP EC, *PTEN* loss and mutations in *PIK3CA* are observed in 77% and 53% of cases, respectively.^9^ Given the cross-regulation between ER and PI3K/Akt/mTOR pathways, the efficacy of the mTOR inhibitor everolimus was assessed in combination with letrozole in a phase II study in patients with recurrent EC. They found an ORR of 32% and a clinical benefit rate of 40%.^70^ This combination was then further assessed with the addition of metformin, given metformin use has been shown to down regulate the PI3K/Akt/mTOR pathway in EC.^71^ This regime of everolimus, letrozole and metformin in women with advanced or recurrent EC found an ORR of 28% and clinical benefit of 50%.^71^ A second example is the use of the cyclin-dependent kinase (CDK) 4 and 6 inhibitor palbociclib. Palbociclib has been shown to inhibit growth of ER-positive breast cancer cells, and palbociclib plus letrozole resulted in significantly longer progression-free survival compared with letrozole alone in ER-positive breast cancer.^72^ A phase II study of palbociclib combined with letrozole in patients with ER-positive advanced or recurrent EC was presented at ESMO 2020 (NSGO – PALEO trial). The palbociclib and letrozole combination resulted in a disease control rate of 64% compared with 38% in letrozole only, and a significantly improved progression-free survival (8.3 months *versus* 3 months).^73^

Obesity and diabetes are common in women with EC, especially within patients with NSMP molecular subtype. The efficacy of the antidiabetic drug metformin was assessed in combination with paclitaxel and carboplatin in 469 patients with treatment naïve advanced or recurrent EC (GOG-0286B). Progression-free survival and overall survival were not increased significantly with the addition of metformin to chemotherapy; however, translational studies are ongoing to identify potential biomarkers that may predict response to metformin treatment.^74^

## POLEmut endometrial cancer

*POLE*mut EC is the least common molecular subtype, accounting for approximately 10% of all ECs. These ECs have pathogenic mutations in the exonuclease domain of DNA polymerase epsilon (*POLE*), a protein involved in DNA replication. This results in extremely high somatic mutation frequencies (‘ultramutated’), exceeding 100 mutations per megabase.^9^ Most *POLE*mut ECs are endometrioid and, despite many having high-risk features, such as high-grade and LVI, this molecular subtype has exceptionally favourable survival outcomes.^10–14,28^ One proposed explanation for this excellent prognosis is the host response to tumour. The extreme tumour mutation burden in *POLE*mut ECs was shown to result in a striking CD8+ lymphocytic infiltrate, as well as marked upregulation of cytotoxic T-cell effector markers in a study of 60 *POLE* mutant tumours.^75^ Another possible explanation for favourable outcomes had been that *POLE*mut ECs are hypersensitive to adjuvant treatment. Van Gool *et al.* created *POLE* mutant mouse-derived embryonic stem cell lines and demonstrated *POLE* mutations did not exhibit increased sensitivity to radiation or commonly used chemotherapeutics compared with *POLE*-wildytpe cell lines.^76^ Further, a recent meta-analysis of all published cases of *POLE* mutations in EC showed that the excellent survival outcomes of stage I/II *POLE*mut EC was independent of adjuvant treatment received.^77^ The molecular analysis of PORTEC-3 – a trial comparing chemoradiation *versus* radiation only in high risk EC – again demonstrated the excellent prognosis of *POLE*mut EC, even within this high risk cohort, with 5-year overall survival of 100% with chemoradiation and 97% with radiation alone (one recurrence event).^28^

### De-escalation of adjuvant therapy

There are two currently active clinical trials assessing the safety of de-escalation of adjuvant treatment in *POLE*mut EC (PORTEC-4a and TAPER – tailored adjuvant therapy in *POLE*-mutated and p53-wildtype early stage endometrial cancer). Further, there is a trial under consideration with the NCI Community Oncology Research Program (NCORP) and the imminent TransPORTEC RAINBO Blue-*POLE* trial (Figure 1). The 2020 ESGO/ESTRO/ESP guidelines classify stage I–II *POLE*mut EC as low risk and state that in women with stage I-II pathogenic *POLE*mut ECs, omission of adjuvant therapy should be considered.^22^ The recent *POLE* meta-analysis included 294 patients with pathogenic *POLE* mutations, and showed recurrence rates are extremely low, with only 11 progression/recurrence events and 3 disease-specific deaths in the total group. What is also reassuring is salvage rates for patients that did have a recurrence were high, with 8 out of the 11 still alive with no evidence of disease up to 14 years after treatment.^77^

### Treatment options in advanced or recurrent POLEmut EC

There is currently insufficient evidence to guide conventional adjuvant treatment of advanced stage (III–IV) *POLE*mut EC. The TransPORTEC RAINBO Blue-*POLE* trial will consider enrolment of stage III ECs allowing observation only or radiotherapy for these women. Other than the aforementioned recent meta-analysis and small retrospective series in which adjuvant treatment was given for advanced stage ECs (molecular subtype unknown at time of treatment), there are no data to infer efficacy. For rare advanced or recurrent *POLE*mut ECs, consideration may be given to immune checkpoint blockade therapy given the ‘ultramutated’ phenotype, with high TIL and PD-1/PD-L1 expression levels.^78–82^

### Importance of characterisation of all molecular classification components and management of ECs with more than one molecular feature (‘multiple classifier ECs’)

**Figure 2.**
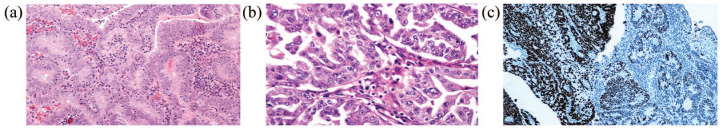
POLEmut endometrial cancer. (a) H&E staining (low power) showing endometrioid features with a prominent host inflammatory infiltrate. (b) H&E staining (higher power) showing an area with serous-like features, including nuclear pleomorphism. The variable morphology, host lymphocytic infiltrate and nuclear atypia are all features associated with POLEmut tumours. In this case there is also subclonal mutant pattern expression of p53 (c), with overexpression on the left and wild-type expression on the right. H&E, haematoxylin and eosin; POLE, polymerase epsilon.

Given the high mutational burden observed in *POLE*mut tumours, secondary *TP53* mutations/p53 IHC abnormalities or less commonly, MMR protein loss can be found. Leon-Castillo *et al.* reported on ECs that have more than one molecular feature.^83^ They demonstrated that tumours with a pathogenic mutation in *POLE* that also have MMRd and/or mutations in *TP53*, have morphology, molecular profiles and clinical behaviour aligning with *POLE*mut EC. These findings suggest that these secondary mutations are a later event acquired during tumour progression that do not affect the clinical outcome. This can sometimes be suggested based on histopathological examination, for example, the presence of (multifocal) subclonal mutant-like IHC expression of p53 (Figure 2). This also highlights the importance of interpreting p53 and MMR IHC in the context of *POLE* mutation status to avoid overtreatment. With the 2020 ESGO/ESTRO/ESP guidelines for the management of patients with endometrial carcinoma, any stage p53abn EC with myometrial invasion is considered high risk and is recommended adjuvant chemotherapy with or without radiation. A stage I–II *POLE*mut EC however is considered low-risk and omission of adjuvant therapy should be considered.^22^ Clarification of pathogenicity of *POLE* mutation is also imperative, with pathogenic list of mutations considered ‘actionable’ for *POLE*mut assignment currently limited to 11 well characterised missense mutations.^84^ Assessing clinical outcomes in women with mutations limited to the list of 11 pathogenic *POLE* mutations *versus* other non-pathogenic mutations in the recently assembled meta-analysis cohorts demonstrated statistically significant differences in outcomes.^77^ Women with non-pathogenic *POLE* mutations should not be offered de-escalation of adjuvant therapy.

## Summary

Molecular classification of EC offers an objective and reproducible classification system that has important prognostic and therapeutic implications. Molecularly directed EC clinical trials must be a priority to enable assessment of treatment efficacy within biologically ‘like’ tumours and to enable improvements in outcomes in this disease site. As we move forward in this new era, further stratification within each molecular subtype will likely refine our application of systemic therapy in EC. It is now recommended that molecular classification should be considered in all ECs and be incorporated into management decisions.
